# Insecticidal Activity of Extracts, Fractions, and Pure Molecules of *Cissampelos pareira* Linnaeus against Aphid, *Aphis craccivora* Koch

**DOI:** 10.3390/molecules27030633

**Published:** 2022-01-19

**Authors:** Surekha Kumari, Shudh Kirti Dolma, Anmol, Upendra Sharma, S. G. Eswara Reddy

**Affiliations:** 1 Chemical Technology Division, CSIR-Institute of Himalayan Bioresource Technology, Palampur 176061, Himachal Pradesh, India; surekhakumari46@gmail.com (S.K.); sanmol472@gmail.com (A.); 2 Academy of Scientific and Innovative Research (AcSIR), CSIR-HRDC Campus, Ghaziabad 201002, Uttar Pradesh, India; skdolma@gmail.com; 3 Entomology Laboratory, Agrotechnology Division, CSIR-Institute of Himalayan Bioresource Technology, Palampur 176061, Himachal Pradesh, India

**Keywords:** *Cissampelos pareira*, isoquinoline alkaloids, bioassay, aphid

## Abstract

*Aphis craccivora* Koch is a polyphagous and major pest of leguminous crops causing significant damage by reducing the yield. Repeated application of synthetic insecticides for the control of aphids has led to development of resistance. Therefore, the present study aimed to screen the insecticidal activity of root/stem extracts/fractions, and pure molecules from *Cissampelos pareira* Linnaeus against *A. craccivora* for identification of lead(s). Among root extract/fractions, the *n*-hexane fraction was found most effective (LC_50_ = 1828.19 mg/L) against *A. craccivora*, followed by parent extract (LC_50_ = 2211.54 mg/L). Among stem extract/fractions, the *n*-hexane fraction (LC_50_ = 1246.92 mg/L) was more effective than the water and *n*-butanol fractions. Based on GC and GC-MS analysis, among different compounds identified in the *n*-hexane fraction of root and stem, ethyl palmitate (known to possess insecticidal activity) was present in the highest concentration (24.94 to 52.95%) in both the fractions. Among pure molecules, pareirarineformate was found most effective (LC_50_ = 1491.93 mg/L) against *A. craccivora*, followed by cissamine (LC_50_ = 1556.31 mg/L). Parent extract and fractions of *C. pareira* possess promising activity against aphid. Further, field bio-efficacy studies are necessary to validate the current findings for the development of botanical formulation.

## 1. Introduction

*Aphis craccivora* Koch (Hemiptera: Aphididae) is one of the most common polyphagous pest [1] reported in 50 host plants (19 families) and is considered as global threat of leguminous plants [2,3]. The nymphs and adults suck the sap from leaves, flowers, and pods of cowpea plants. The aphid also transmits plant viruses [4,5] and affects the yield [6]. In severe infestation, *A. craccivora* secrete honeydew on the plants, which serves as a medium for the growth of sooty mold, there by leaves became black and affect photosynthesis [7] and reported significant reduction in the seed yield to the extent of 12.8 to 61.1% [8].

The plant extracts and their formulations are normally less harmful to the environment, have low cost, and are less persistent, and safer to natural enemies and humans, and easily biodegradable than synthetic insecticides [9,10]. Numerous studies have already been done on plant-derived extracts/essential oils and their isolated compounds against insect pests [11]. Due to less availability of biopesticides, farmers/growers often spray synthetic insecticides (imidacloprid, thiamethoxam, acetamiprid, thiacloprid, diafenthiuron, chlorfenapyr, spiromesifen, and dimethoate) to control aphids [12,13], and other insecticides, which led to the development of insect resistance [14,15], and are harmful to the environment, non-target insects, and human health [16].

*Cissampelos pareira* Linn. (Menispermaceae) is a climber commonly called Ambasthaor Laghupatha in Ayurveda, distributed in the tropical and subtropical parts of India. The plant is traditionally used against numerous ailments such as indolent ulcers, cholera, diarrhea, asthma, rheumatism, epilepsy, fever, dysentery, and rabies [17,18]. In addition, the root paste is applied topically on wounds, snakebite, dog bite, and skin rashes [19]. The decoctions/infusions (aqueous or alcoholic) of roots and leaves are traditionally used to treat malaria, pneumonia, and as snake anti-venom [20,21]. *C. pareira* plants also have several diverse pharmacological activities [22,23,24,25,26,27].

A literature survey revealed that this plant is reported to possess isoquinoline alkaloids as major compounds. Isoquinolines represent the unique structural motifs in several biologically active natural and synthetic products. There are various reports on the insecticidal activity of isoquinoline type molecules and plants possessing isoquinoline alkaloids [28]. Although this plant is documented for its effect on stored grain pests [29], larvicidal activity against mosquitoes [30], and antimalarial properties of root against *Plasmodium falciparum* and *P. berghei* [31,32,33], but there has been no report on its insecticidal activities against crop pests including *A. craccivora*. Hence, on the basis of type of phytochemicals present in plant, the main objective of this study was to evaluate plant extracts/fractions and compounds (isoquinoline compounds) from *C. pareira* for their insecticidal activity against *A. craccivora* and to identify the lead(s) for botanical formulation for the control of aphids.

## 2. Results

### 2.1. Characterization of Isolated Molecules

The chemical structures of isolated molecules were elucidated by nuclear magnetic resonance spectroscopy (NMR), HRESI-MS, and finally by comparison with those reported in the literature [33,34]. Proton and carbon NMR spectra are shown in the supplementary information.

Curine (**1**): White amorphous powder, *m. p.* 298–300 °C; UV/Vis (1N HCl) λ_max_ (log ε) 292.5 (2.034) nm; IR (ZnSe) ν_max_3514, 2949, 1612, 1504, 1438, 1226, 1111, 831, 638, 582 cm^−1^; ^1^H-NMR (methanol-*d*_4_ + acetic acid- *d*_4_ 600 MHz) δ 7.45 (d, *J* = 8.0 Hz, 1H, H-14′), 7.32 (d, *J* = 7.7 Hz, 1H, H-14), 7.05 (s, 1H, H-5),7.02 (d, *J* = 8.3 Hz, 1H, H-13) 6.91 (d, 1H, H-13′), 6.90 (s, 1H, H-5′), 6.63 (d, *J* = 7.9 Hz, 1H, H-10′),6.56 (d, *J* = 6.9 Hz, 1H, H-11′), 6.46 (s, 1H, H-10), 5.55 (s, 1H, H-8′), 4.91 (s, 1H, H-1), 4.54 (d, 1H, H-1′), 3.98 (s, 3H, 6-OCH_3_), 3.96 (s, 3H, 6′-OCH_3_), 3.83 (m, 1H, H-3′), 3.56–3.53 (m, 1H, H-3′), 3.56–3.54 (m, 2H, H-15′), 3.46–3.44 (m, 1H, H-3), 3.46–3.44 (m, 1H, H-15), 3.26–3.21 (m, 1H, H-4′), 3.11–3.08 (m, 1H, H-4′), 3.03 (s, 3H, 2′(N-CH_3_), 2.95–2.90 (m, 1H, H-3), 2.95–2.90 (m, 1H, H-15), 2.98 (s, 3H, 2(N-CH_3_), 2.95–2.90 (m, 1H, H-4), 2.73 (m, 1H, H-4). ^13^C-NMR (methanol-*d*_4_ + acetic acid- *d*_4_ 150 MHz) δ 156.1 (C-12′), 149.1 (C-6′), 149.0 (C-6), 147.8 (C-12), 145.0 (C-7′), 141.7 (C-11), 138.3 (C-8), 137.6 (C-7), 131.4 (C-10′), 129.5 (C-14′), 128.8 (C-9′), 128.7 (C-9), 123.7 (C-14), 123.6 (C-4a’), 122.3 (C-10), 121.8 (C-8a’), 120.8 (C-8a), 119.9 (C-4a), 116.7 (C-13), 116.0 (C-13′), 115.2 (C-8′), 113.6 (C-11′), 112.6 (C-5′), 108.2 (C-5), 63.5 (C-1′), 58.2 (C-1), 55.9 (6-OCH_3_), 55.6 (6′-OCH_3_), 44.8 (C-3), 44.4 (C-3′), 39.9 (2(N-CH_3_), 39.4 (2′(N-CH_3_), 38.3 (C-15), 38.2 (C-15′), 21.9 (C-4′), 21.9 (C-4) (Figure S1a,b; Table S1); HRESI-MS: [M + H]^+^ 595.2754 [34].

Pareirarineformate (**2**): Brown solid; *m.p.*262 ° C; UV/Vis (EtOH) λ_max_ (log ε) 289 (1.194) nm; IR (ZnSe) ν_max_ 3377, 2918, 2448, 1597, 1440, 1357, 1230, 1112, 1020, 862 cm^−1^;^1^H-NMR (600 MHz, Methanol-*d*_4_) δ 6.85 (d, *J* = 8.2 Hz, 1H, H-5′), 6.83 (s, 1H, H-5), 6.51 (d, *J* = 2.1 Hz, 1H, H-2′), 6.48 (dd, *J* = 8.2, 2.1 Hz, 1H, H-6′), 5.73 (s, 1H, H-8), 4.60 (dd, 1H, H-1), 3.90–3.97 (m, 1H, H-3), 3.82 (s, 3H, OCH_3_), 3.80 (s, 3H, OCH_3_), 3.61–3.63 (m, 1H, H-3), 3.58–3.60 (m, 1H, H-9), 3.43 (s, 3H, (2(N-CH_3_), 3.37 (s, 3H, OCH_3_), 3.19–3.22 (m, 2H, H-4), 3.15 (s, 3H, (2(N-CH_3_), 2.80–2.84 (m, 1H, H-9).^13^CNMR (150 MHz, methanol-*d*_4_) δ 150.9 (C-6), 148.5 (C-4′), 148.4 (C-3′), 147.1 (C-7), 129.3 (C-1′), 123.1 (C-8a), 122.6 (C-6′), 121.8 (C-4a), 118.3 (C-2′), 113.2 (C-8), 112.9 (C-5′), 112.4 (C-5), 74.1 (C-1), 56.5 (OCH_3_), 56.3 (OCH_3_), 55.9 (OCH_3_), 55.7 (C-3), 52.9 (2(N-CH_3_), 51.3 (2(N-CH_3_), 38.4 (C-9), 24.4 (C-4) (Figure S2a,b; Table S2); HRESI-MS: [M]^+^358.2056 [32].

Cissamine (**3**): Yellow powder; *m.p*. 220–222 °C; UV/Vis (EtOH) λ_max_ (log ε) 289.5 (1.666) nm; IR (ZnSe) ν_max_3523, 2337, 1531, 1504, 1442, 1249, 1087, 875 cm^−1^;^1^H-NMR (Methanol-*d*_4_, 600 MHz) δ 6.95 (dd, *J* = 8.4, 2.0 Hz, 1H, H-11), 6.87 (s, 1H, H-4), 6.72 (s, 1H, H-1), 6.71 (d, 1H), 4.81–4.85 (m, 1H, H-8), 4.69–4.71 (m, 1H, H-13a), 4.62 (s, 1H, H-8), 3.86 (s, 3H, 3-OCH_3_), 3.85 (s, 3H, 10-OCH_3_), 3.56–3.60 (m, 1H, H-6), 3.37–3.42 (m, 1H, H-6), 3.29 (m, 1H, H-13), 3.27 (s, 3H, 7(N-CH_3_), 3.22–3.26 (m, 1H, H-5), 3.11–3.16 (m, 1H, H-13).^13^C-NMR (150 MHz, methanol-*d*_4_) δ 149.9 (C-3), 147.5 (C-10), 147.2 (C-2), 144.4 (C-9), 125.3 (4a), 123.3 (C-12a), 120.4 (C-8a), 119.8 (C-12), 114.3 (C-1), 113.1 (C-4), 113.0 (C-11), 67.0 (C-13a), 60.6 (C-8), 56.6 (3-OCH_3_), 56.5 (10-OCH_3_), 53.8 (C-6), 50.9 (7(N-CH_3_), 34.6 (C-13), 24.1 (C-5) (Figure S3a,b; Table S3); HRESI-MS: [M]^+^ 342.1710 [32].

### 2.2. Quantification of Isolated Molecules

Isolated molecules namely curine (**1**), pareirarineformate (**2**), and cissamine (**3**) were quantified by UPLC-DAD method in different extracts and fractions of *C. pareira* (Table 1). Quantification results clearly depicted that the above-mentioned isoquinoline alkaloids were present in almost all extracts and fractions, albeit in variable quantities.

### 2.3. GC-MS Analysis of n-Hexane Fractions of Root and Stem

Methylation of *n-*hexane fraction was carried out to convert the fatty acid into corresponding methyl ester derivatives which were further analyzed with GC and GC-MS. Total seven compounds were identified including methyl hexadecanoate (24.94% in root fraction and 52.95% in stem fraction), methyl 8-octadecenoate (36.76% in root fraction), methyl 9-octadecenoate (30.37% in root and 10.53% in stem fraction), and methyl octadeca-9,12-dienoate (2.60% in root and 9.24% in stem) (Table 2).

### 2.4. Bio-Assay of Extract, Fractions, and Pure Molecules of C. pareira against A. craccivora

Parent extracts and fractions of *C. pareira* root were evaluated for their efficacy against *A. craccivora* under laboratory conditions (Table 3 and Figure S4). Both parent extract and its fractions were found effective against *A. craccivora*. At 72 h after treatment, the *n*-hexane fraction (LC_50_ = 2817.52 mg/L) showed more promising activity against aphid followed by *n*-butanol fraction (LC_50_ = 5614.74 mg/L), chloroform fraction (LC_50_ = 5983.62 mg/L), parent extract (LC_50_ = 6295.49 mg/L), water fraction (LC_50_ = 8848.12 mg/L), and water extract decoction (root) (LC_50_ = 8361.78 mg/L). However, all the extracts and fractions of *C. pareira* were not superior to Neem Baan 0.15% EC (positive control) after 72 h (2587.32 mg/L). At 96 h after treatment, *n-*hexane fraction (LC_50_ = 1828.19 mg/L) was found more effective followed by parent extract (LC_50_ = 2211.54 mg/L), *n*-butanol fraction (LC_50_ = 3153.47 mg/L), chloroform fraction (LC_50_ = 3254.76 mg/L), water fraction (LC_50_ = 7168.12 mg/L), and water extract decoction (root + stem) (LC_50_ = 8848.12 mg/L). However, parent extract and fractions of *C. pareira* were not superior to Neem Baan 0.15% EC after 96 h (LC_50_ = 1206.44 mg/L).

With respect to mortality, the *n*-hexane fraction at a higher concentration (10,000 mg/L) was significantly (F_4,14_ = 45.14; *p* < 0.0001) more effective (83.33% mortality) against *A. craccivora* followed by chloroform (70%), the *n*-butanol (66.67%) fraction as compared to parent extract (56.67%), and water fraction (56.67%). Similarly, at 96 h after treatment, the *n*-hexane fraction was significantly (F_4,14_ = 62.50; *p* < 0.0001) more effective (100%) followed by parent extract (93.33%) and chloroform fraction (93.33%) as compared to the water fraction (66.67%). The parent extract, *n*-hexane, and chloroform fraction were at par with the positive control (Neem Baan at 5000 mg/L) (Figure S4.).

Parent extract and fractions of *C. pareira* stem were evaluated for their efficacy against *A. craccivora* under laboratory conditions (Table 4 and Figure S5). Both parent extract and its fractions were found effective against *A. craccivora*. At 72 h after treatment, the *n*-hexane fraction (LC_50_ = 1466.98 mg/L) showed more promising activity against aphids followed by ethyl acetate (LC_50_ = 5534.27 mg/L), water fraction (LC_50_ = 5861.38 mg/L), parent extract (LC_50_ = 5929.00 mg/L), and the *n*-butanol fraction (LC_50_ = 7766.81 mg/L). At 96 h after treatment, the *n-*hexane fraction (LC_50_ = 1246.92 mg/L) was found more effective against aphids followed by water (LC_50_ = 3761.37 mg/L), *n-*butanol (LC_50_ = 3840.96 mg/L), ethyl acetate fraction (LC_50_ = 3992.4 mg/L), and parent extract (LC_50_ = 4044.83 mg/L). However, all the extracts and fractions of *C. pareira* were not superior to Neem Baan after 72 and 96 h (LC_50_ = 2587.32 and 1206.44 mg/L, respectively) except for the *n*-hexane fraction (LC_50_ = 1466.98 mg/L) after 72 h.

With respect to mortality, the *n*-hexane fraction at a higher concentration (10,000 mg/L) was significantly (F_4,14_ = 82.50; *p* < 0.0001) more effective (96.67% mortality) against *A. craccivora* followed by parent extract (70%), ethyl acetate (70%) fraction as compared to *n*-butanol (60%), water fraction (56.67%), and water fraction decoction root (53.33%). Similarly, at 96 h after treatment, the *n*-hexane fraction was significantly (F_4,14_ = 130.63; *p* < 0.0001) more effective (100%) followed by parent extract (86.67%), ethyl acetate fraction (86.67%), and *n*-butanol (83.33%) as compared to the water fraction and its decoctions (73.33%). Among the fractions and parent extracts, the *n*-hexane fraction was at par with the positive control, i.e., Neem Baan at 5000 mg/L (96.67%) (Figure S5).

The molecules namely curine (**1**), pareirarineformate (**2**), and cissamine (**3**) of *C. pareira* were evaluated for their efficacy against *A. craccivora* under laboratory conditions and were found effective against aphid at 48 and 72 h after treatment (Table 5 and Figure S6). At 48 h after treatment, pareirarineformate (**2**) (LC_50_ = 1860.57 mg/L) was found more effective against *A. craccivora* followed by cissamine (**3**) (LC_50_ = 2744.95 mg/L). Similarly, at 72 h after treatment, pareirarineformate (**2**) (LC_50_ = 1491.93 mg/L) was found more effective against *A. craccivora* followed by cissamine (**3**) (LC_50_ = 1556.31 mg/L) and curine (**1**) (LC_50_ = 3802.47 mg/L). However, all the molecules of *C. pareira* were found superior to Neem Baan 0.15% EC after 72 h (LC_50_ = 2587.32 mg/L) except curine.

With respect to mortality, among the compounds pareirarineformate (**2**) at 5000 mg/L was significantly (F_4,14_ = 51.56; *p* < 0.0001) more effective (83.33% mortality) against *A. craccivora* after 48 h of treatment followed by cissamine (**3**) (70%) as compared to curine (**1**) (43.33%) which showed less mortality. The pareirarineformate (**2**) was superior to the positive control, whereas the cissamine (**3**) was at par with the positive control, i.e., Neem Baan at 5000 mg/L (70%). Similarly, pareirarineformate (**2**) was significantly (F_4,14_ = 48.50; *p* < 0.0001) more effective (93.33%) against *A. craccivora* after 72 h of treatment followed by cissamine (**3**) and curine (**1**) (90% and 83.33%, respectively). The pareirarineformate (**2**) and cissamine (**3**) were at par with Neem Baan (96.67%) (Figure S6.).

## 3. Discussion

Despite using chemical pesticides, there is considerable evidence indicating the loss of agriculture production and the potential threat of these chemical pesticides on humankind and biodiversity. Similarly, the resistance shown by insect pest is also a major concern, which emphasizes looking for alternative bio-tools for which role of plants comes into play. Plants have potential to serve as a greener alternative to chemical pesticides which is further proved by evaluating the insecticidal potential of *C. pareira* which showed promising activity against *A. craccivora*. Parent extracts, their fractions, and pure molecules of *C. pareira* against *A. craccivora* were tested. In the present study, the *n-*hexane fractions of root and stem were found more effective against *A. craccivora* followed by the parent extract of root, *n*-butanol, and chloroform fraction compared to stem fractions of water and *n*-butanol. The*n-*hexane fraction (LC_50_ = 1828.19 mg/L) and parent extract of *C. pareira* root (LC_50_ = 2211.54 mg/L) were more effective against *A. craccivora* in this study as compared to *n*-hexane fraction of *Eupatorium adenophorum* (LC_50_ = 2881 mg/L) and *Ageratum houstonianum* (LC_50_ = 2590 mg/L) [41,42]. GC analysis of *n*-hexane fractions of both root and stem confirmed the presence of esters of fatty acids. The *n*-hexane fraction of stem has high content of ethyl palmitate (52.95%), ethyl oleate (10.53%), and methyl linoleate (9.24%), whereas the *n*-hexane fraction of the root contains high contents of 8-octadecenoic acid (36.76%), ethyl oleate (30.37%), and ethyl palmitate (24.94%). These compounds are already known for their insecticidal potential. Ethyl oleate and ethyl palmitate were found in the extract of *Eupatorium odoratum* which act as oviposition repellent [43,44]. Ethyl palmitate is reported to have larvicidal activity [45,46]. Therefore, the presence of these compounds in *n*-hexane fractions of root and stem could be attributed to the potent insecticidal activity against *A. craccivora*.

In a similar study, the *n*-hexane fraction from tubers of *Corydalis turtschaninovii* at 2000 mg/L showed less efficacy (85% mortality) against *Aphis gossypii* [28] as compared to the present study. In another study, the ethanol root extract of *Cissampelosmu cronata* found more effective against larvae of *Culex quinquefasciatus* (LC_50_ = 207.1 μg/mL) after 72 h [27] as compared to the present study. Similarly, in this study, the ethanol stem extract of *C. pareira* at a lower concentration (LC_50_ = 1466.98 mg/L) was more effective against *A. craccivora* after 72 h as compared to aerial parts of ethanol extract of *C. mucronata* (LC_50_ = 8000 μg/mL) against larvae of *C. quinquefasciatus* after 72 h [27]. In another study *C. awariensis* leaf and root slurries at 1% showed 96–100% mortality against *Prostephanustruncatus* horn and *Sitophilus oryzae* L. [29] as compared to the present study where *C. pareira* root and stem extract showed more promising activity at a lower dose.

Insecticidal activity of parent extract/fractions from the root and stem of *C. pareira* against *A. craccivora* might also be due to the presence of alkaloids/molecules (curine, pareirarineformate, and cissamine). Compounds pareirarineformate (**2**) (LC_50_ = 1491–1860 mg/L) and cissamine (**3**) (LC_50_ = 1556–2744 mg/L) were effective against *A. craccivora* after 48 and 72 h as compared to curine (**1**) (LC_50_ = 3802 mg/L). Insecticidal activity results were further validated by quantification data showing the presence of high alkaloid content in the *n*-butanol fraction of roots from where pareirarineformate (**2**) and cissamine (**3**) are isolated. Thus, the presence of isoquinoline type molecules could be the reason for the high activity of *n*-butanol fraction of *C. pareira* against *A. craccivora*. In comparison with other studies, pareirarineformate (**2**) (LC_50_ = 1491 mg/L) and cissamine (**3**) (LC_50_ = 1556 mg/L) were more active against *A. craccivora* after 48 h as compared to 1-(3-nitrophenyl)-6,7-dimethoxy tetrahydro isoquinoline (LC_50_ = 1624 µg mL^−1^) against larvae of *C. quinquefasciatus* [47] but less effective than 1-(4-methoxy) and 1-(4-chlorophenyl)-6,7-dihydroxytetrahydroisoquinoline (LC_50_ = 179–479 µg mL^−1^). In another study, the compounds/alkaloids viz., dimethyl corydalmine, (+)-stylopine, isocorypalmine, pseudoprotopine, and glaucine at 1000 mg/l from tubers of *Corydalis turtschaninovii* showed less mortality (68% to 80%) against *Aphis gossypii* after 72 h [28] as compared to pareirarineformate (**2**) (LC_50_ = 1491 mg/L) and cissamine (**3**) (LC_50_ = 1556 mg/L) against *A. craccivora* in the present study. The present study concludes that *C. pareira* parent extracts, fractions, and molecules (pareirarineformate and cissamine) can be used for the management of aphid subject to field bio-efficacy studies.

## 4. Materials and Methods

### 4.1. Collection and Authentication of Plant Material

The root and stem part of *C. pareira* were collected from Palampur, Himachal Pradesh, India in January 2019. The plant material was authenticated by a taxonomy expert at CSIR-IHBT and submitted to the herbarium of CSIR-IHBT, Palampur with voucher specimen no. PLP16688. Plant material was shade-dried for about a week and then ground into uniform powder using a MAC Willy mill PLT 210 grinder.

### 4.2. Preparation of Extracts, Fractions and Decoctions

**Figure 1:**
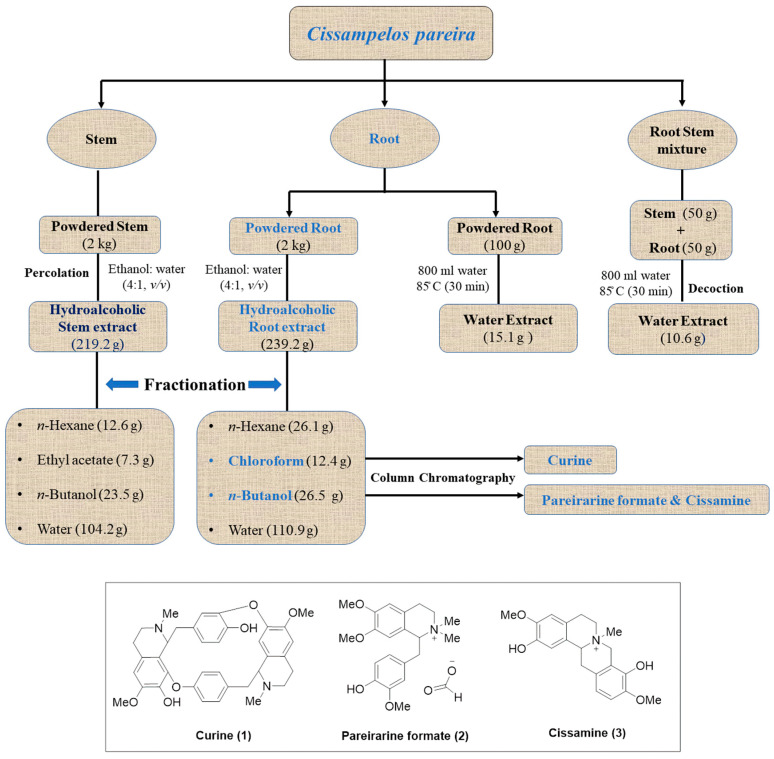
Schematic diagram showing complete steps involved in extraction, fractionation, and isolation of molecules from *Cissampelos pareira*.

Shade-dried 2-kg powdered roots were extracted thrice with ethanol:water (4:1, *v*/*v*) using the percolation method at room temperature. The percolate collected from the extraction was evaporated in a rotary evaporator at 50 °C to obtain 239.2 g of hydro-ethanolic crude extract. This dried crude extract was dissolved in 700 mL of distilled water, and after that the dissolved part was further fractionated with organic solvents (600 mL × 3 times), i.e., *n-*hexane, chloroform, and *n-*butanol to yield fractions of different polarity i.e., *n*-hexane (26.1 g), chloroform (12.4 g), *n*-butanol (26.5 g), and water (110.9 g) (Figure 1). Similarly, the stem part (2 kg) was processed by the above procedure to obtain crude extract 219.2 g and fractions as follows: *n*-hexane (12.6 g), ethyl acetate (7.3 g), *n-*butanol (23.5 g), and water (104.2 g) (Figure 1). Decoctions were prepared by boiling the plant material with distilled water at a temperature of 85 °C for 30 min. Root decoction was prepared by boiling the crushed roots (100 g) with 800 mL of distilled water. Then the decoction was concentrated on a rotary evaporator at a temperature of 50 °C to obtain 15.1 g of the water extract. The combined decoction of roots (50 g) and stem (50 g) was prepared in a similar way to obtain 10.6 g of the water extract (Figure 1).

### 4.3. Isolation of Pure Molecules

The chloroform fraction (12.0 g) of roots was chromatographed on silica gel (60–120 mesh) and eluted using a CH_3_OH:CHCl_3_ mixture (0.0:10, 0.5:9.5, 1.0:9.0, 1.5:8.5, 2.0:8.0, 2.5:7.5, 3.0:7.0, 5.0:5.0, and 10:0.0 *v*/*v*) as the mobile phase. A total of fifteen fractions were collected. From fraction 11 (collected at polarity 2.0:8.0), the compound curine (**1**) (190 mg) was precipitated out when left undisturbed overnight. The precipitated compound and the supernatant were checked by TLC analysis based on which the precipitated part was identified as the pure compound.

The *n*-butanol fraction (25.0 g) of roots was subjected to chromatography over silica gel (60–120 mesh) and eluted with increasing gradient of CH_3_OH:CHCl_3_(0.0:10, 0.5:9.5, 1.0:9.0, 1.5:8.5, 2.0:8.0, 2.5:7.5, 3.0:7.0, 5.0:5.0, and 10:0.0 *v*/*v*). A total of twenty-one fractions were collected. Fraction 13 (4.1 g) was chromatographed over silica gel (230–400 mesh) using CH_3_OH:CHCl_3_ (1.0:9.0, 1.5:8.5, 2.0:8.0 *v*/*v*) to afford four sub-fractions. Then sub-fraction 2 (1.6 g) was further subjected for chromatography on silica gel (230–400 mesh) using CH_3_OH:CHCl_3_ (2.0:8.0 *v*/*v*) with 5 mL of formic acid to afford (**2**) as pareirarineformate (0.137 g). Fraction 14 (3.4 g) was further chromatographed over silica gel (230–400 mesh), and CH_3_OH:CHCl_3_ (1.5:8.5 to 5.0:5.0) was used as mobile phase. Five sub-fractions were collected from fraction 14. Sub-fraction 2 (0.746 g) was further chromatographed over silica gel (230–400 mesh), eluted with CH_3_OH:CHCl_3_ (1.5:9.5 to 2.5:7.5), and two fractions were collected. Then from sub-fraction 1 (0.546 g), cissamine (**3**) (0.271 g) was isolated by column chromatography over silica gel (230–400 mesh) and eluted with CH_3_OH:CHCl_3_ (2.0:8.0 *v*/*v*). The chemical structures of these isolated compounds are shown in Figure 1.

### 4.4. Characterization of Molecules Isolated from C. pareira

All the molecules isolated from *C. pareira* were characterized by ^1^H-NMR, ^13^C-NMR, UV/Vis, and IR spectroscopy. The melting points of all the molecules were noted on Brønsted Electro thermal 9100. NMR spectral analysis of these molecules was done on Bruker-Avance 600 MHz instrument. UV–Vis analysis was performed on Shimadzu UV-VIS spectrometer-2600. IR analysis was done on Shimadzu IR Prestige-21with ZnSe single reflection ATR accessory.

### 4.5. Preparation of Methyl Esters

The *n*-hexane fractions of root (50.1 mg) and stem (52.1 mg) were derivatized using methanol and sulfuric acid under nitrogen atmosphere [48]. The derivatized fractions thus obtained were evaluated by gas chromatography (GC-FID and GC-MS).

### 4.6. Quantification of Compounds in Extract, Fractions and Decoctions of C. pareira

The quantification of marker compounds (**1**, **2**, and **3**) in different extracts (root and stem) and fractions as well as decoctions of root and stem part of *C. pareira* was performed by UPLC-DAD method reported earlier by our group [33]. Chromatograms for quantification of compounds in extract, fractions, and decoctions are shown in Supplementary Figure S7a–d.

### 4.7. GC-FID Analysis of n-Hexane Fractions

The *n*-hexane fractions were subjected to GC-FID analysis using GC Shimadzu 2010 coupled with AOC-20i auto-injector, SH-Rxi-5Sil MS column (30 m × 0.25 mm i.d., 0.25 μm) and FID-detector. Nitrogen was used as a carrier gas with a flow rate of 1.24 mL/min. The initial temperature of oven was 40 °C for 4 min and programmed to 220 °C at 4 min, then held for 15 min at 220 °C. Other parameters for GC analysis were an injector temperature of 250 °C, oven temperature of 250 °C, and the split mode was used. A standard solution of *n*-alkanes (C_9_–C_23_) was used to obtain the retention indices. Individual components were identified by matching their retention indices (RI) with those reported in the literature.

### 4.8. Gas Chromatography-Mass Spectrometry Analysis

The GC-MS analysis was carried out on a Shimadzu (GC 2010) GC-MS equipped with an AOC-5000 auto-injector coupled and an SH-Rxi-5Sil MS capillary column (30 m × 0.25 mm i.d., 0.25 μm). The initial temperature of the column was 40 °C held for 4 min and was programmed to 220 °C at 4 min, then held for 21 min at 220 °C; the sample injection volume was 1 μL in the HPLC-grade dichloromethane. Helium was used as carrier gas at a flow rate of 1.28 mL min^−1^ on the split mode (1:10). Individual components were identified by matching their mass spectra with literature, NIST database, and Adams’s libraries [49,50]. GC-MS chromatograms for *n*-hexane fractions of root and stem are shown in Supplementary Figure S8.

### 4.9. Test Insect

*Aphis craccivora* collected on leguminous plants in the field and reared under controlled conditions (26 ± 2 °C temperature, 60 ± 5% humidity, and photoperiod 16:8 L:D) in the lab on the live host (*Phaseolus vulgaris* L.) more than 100–120 generations. The uniformly sized nymphs of 3–4 days old aphid were used for bioassay study.

### 4.10. Dose Optimization

Preliminary screening of root/stem extracts and their fractions was carried out at 5000 and 10,000 mg/L) for their bio-efficacy against *A. craccivora*. Five concentrations were fixed and assessed against aphids in the main bioassay studies based on preliminary efficacy data.

### 4.11. Bioassay of Extracts, Fractions, and Pure Molecules of C. pareira against A. craccivora

Briefly, test samples were dissolved in Triton X 0.05% solution (SD Fine Chemicals Limited, Mumbai, India) in water and then ultrasonicated for complete dissolution. Five concentrations of root extracts/fractions (625 to 10,000 mg/L) and pure compounds (313 to 5000 mg/L) were prepared from stock solutions by serial dilution for dose response bioassay. Fresh bean discs (3 cm diameter) were prepared and pressed over the water–agar medium (1.5%) in Petri plates sprayed with 2 mL of the test solution at different concentrations under Potter’s spray tower operated at 1.1 kg/cm^2^ pressure and the solvent was evaporated under room temperature for 2 h. For control, leaf disks were sprayed with distilled water containing Triton. In each Petri dish, 10 nymphs were released then sealed with parafilm and kept in the laboratory conditions at 25 ± 2 °C temperature, 60 ± 5% relative humidity, and a photoperiod of 16:8 (L:D) for observations. All the treatments including control were replicated three times. Mortality was determined after 72 and 96 h of treatment. There were five treatments and three replications (5 × 30 = 150 insects, each replication contains 10 insects. The commercially available neem formulation (Neem Baan 0.15 EC, i.e., containing azadirachtin 1500 ppm) available in the market (manufactured by Pest Control India Pvt. Limited, Goa, India) used by the farmers/growers at the recommended dose (5 mL/L of water) for the control of aphid on crop plants was used as a positive control for comparison.

### 4.12. Data Analysis

The mortality data of aphid based on bioassays of extracts/fractions/compounds was compiled. Corrected mortality was not calculated because the test insect nymphs were not died in the untreated control. Lethal concentration to kill 50% test population (LC_50_ values) and regression parameters were worked out by Probit analysis [51] using SPSS software version 16. Similarly, the percent mortality data against test insect was also analyzed using analysis of variance (ANOVA), and means were compared by Tukey’s post hoc test [52].

## 5. Conclusions

The present study concludes that parent extract and fractions of *C. pareira* possess promising activity against aphids which can serve as potential biopesticide, as currently used chemical pesticides are being prone to resistance by insect pest along with their adverse environmental hazards. Hence lab-scale bioactivity evaluation can serve as a potential footstep for advanced studies in the demand of plants-based bio-pesticides. However, field bio-efficacy studies are necessary to validate the current findings against target pest for the development of botanical formulation.

## Figures and Tables

**Table 1:** Amount (mg/g ± SD) of compounds in extracts, fractions, and decoctions of root and stem of *Cissampelos pareira*.

Samples	Curine (1)	Pareirarine Formate (2)	Cissamine (3)	Total Alkaloids
USCPR-PE	27.1 ± 0.32	19.6 ± 0.11	54.9 ± 0.32	101.6
USCPR-HF	5.9 ± 0.17	3.0 ± 0.05	7.9 ± 0.05	16.8
USCPR-CF	15.8 ± 0.51	5.9 ± 0.17	10.3 ± 0.30	32.0
USCPR-BF	10.8 ± 0.05	47.8 ± 0.23	167.1 ± 0.66	225.7
USCPR-WF	31.8 ± 0.20	16.5 ± 0.05	39.5 ± 0.23	87.8
USCPS-PE	-	9.0 ± 0.05	5.1 ± 0.14	14.1
USCPS-HF	-	2.7 ± 0.05	1.7 ± 0.06	4.4
USCPS-EA	-	-	-	-
USCPS-BF	-	11.5 ± 0.20	7.3 ± 0.005	18.8
USCPS-WF	-	3.2 ± 0.02	-	3.2
USCPR-W-1	2.9 ± 0.17	12.4 ± 0.30	7.9 ± 0.26	23.2
USCPRS-W-2	2.1 ± 0.05	10.6 ± 0.25	9.6 ± 0.10	22.3

USCPR-PE: parent root extract; USCPR-HF: *n*-hexane fraction of root: USCPR-CF: chloroform fraction of root: USCPR-BF: *n*-butanol fraction of root: USCPR-WF: water fraction of root: USCPS-PE: parent stem extract: USCPS-HF: *n*-hexane fraction of stem; USCPS-EA: ethyl acetate fraction of stem; USCPS-BF: *n*-butanol fraction of stem; USCPS-WF: water fraction of stem; USCPR-W-1: water decoction of root; USCPRS-W-2: water decoction of root and stem. SD: standard deviation.

**Table 2:** Chemical composition of *n*-hexane fractions from *Cissampelos pareira*.

S. No	Compounds	%		RI^a^	RI^b^	Mass Fragmentations
		Root Fraction	Stem Fraction			
1.	Methyl hexadecanoate (Ethyl palmitate)	24.94	52.95	1911	1921 [35]	270 [M]^+^, 227, 199, 185, 171, 143, 129, 101, 87, 74, 55, 43
2.	Methyl 15-methylhexadecanoate (15-methylpalmitic acid)	-	1.97	2000	1990 [36]	285, 241, 199, 185, 171, 143, 101, 87, 74, 57, 43, 41 27
3.	Methyl octadeca-9,12-dienoate (Methyl linoleate)	-	9.24	2062	2075 [37]	294 [M]^+^, 263, 164, 150, 123, 109, 95, 81, 67, 55, 41, 27
4.	Methyl 8-octadecenoate ((8*E*)-8-octadecenoic acid)	36.76	-	2077	2080 [38]	296 [M]^+^, 264, 222, 194, 180, 166, 137, 98, 84, 74, 69, 55, 41, 27
5.	Methyl 9-octadecenoate (Ethyl oleate)	30.37	10.53	2084	2087 [39]	296 [M]^+^, 264, 222, 180, 166, 137, 123, 98, 87, 74, 69, 55, 41, 27
6.	Methyl *n*-octadecanoate (Stearic acid)	4.80	8.38	2113	2111 [37]	298 [M]^+^, 267, 255, 199, 185, 143,129, 101, 87, 74, 57, 43, 41
7.	Methyl 18-methylnonadecanoate (Isoarachidic acid)	0.19	1.94	2272	2277 [40]	326 [M]^+^, 283, 241, 227, 199, 185, 171, 143, 129, 101, 87, 74, 57, 43

RI^a^ =calculated retention indices; RI^b^ =retention indices from literature; -: absent; % = relative percentages calculated from GC-FID.

**Table 3:** Bio-assay of root extracts and fractions of *Cissampelos pareira* against *Aphis craccivora*.

Extracts and Fractions	LC_50_ (mg/L)	95% Confidence Limits (mg/L)	Slope ± SE	Chi Square	*p* Value
Parent extract (72 h)	6295.49	3912.09–16135.69	1.09 ± 0.27	0.32	0.96
Parent extract (96 h)	2211.54	1625.56–2971.02	1.79 ± 0.29	1.95	0.58
*n*-Hexane fraction (72 h)	2817.52	2029.05–4004.10	1.57 ± 0.28	0.86	0.83
*n*-Hexane fraction (96 h)	1828.19	1410.36–2321.77	2.30 ± 0.33	2.41	0.49
Chloroform fraction (72 h	5983.62	4513.91–8913.16	2.04 ± 0.34	0.54	0.91
Chloroform fraction (96 h)	3254.76	2578.93–4166.11	2.43 ± 0.34	2.17	0.54
*n*-Butanol fraction (72 h)	5614.74	4135.22–8717.88	1.80 ± 0.31	0.13	0.99
*n*-Butanol fraction (96 h)	3153.47	2455.03–4131.42	2.16 ± 0.32	0.64	0.89
Water fraction (72 h)	7139.63	5091.65–12348.96	1.75 ± 0.33	0.86	0.84
Water fraction (96 h)	7168.12	5232.76–11733.65	1.93 ± 0.35	1.53	0.67
Water extract decoction (root) (72 h)	8361.78	5695.00–16605.62	1.62 ± 0.32	0.47	0.92
Water extract decoction (root) (96 h)	4783.90	3573.96–7062.31	1.83 ± 0.31	1.25	0.74
Water extract decoction (root + stem) (96 h)	8848.12	6085.65–17252.00	1.73 ± 0.34	0.41	0.94
Neem Baan (0.15 EC) (72 h)	2587.32	1914.19–3944.86	1.52 ± 0.25	0.71	0.87
Neem Baan (0.15 EC) (96 h)	1206.44	953.26–1528.79	1.98 ± 0.26	3.90	0.26

*n* = 150 insects; no. of replications-3; LC_50_ = lethal concentration to kill 50% of test insect; LC_50_ values calculated for extract and fractions showing > 50% mortality using probit analysis.

**Table 4:** Bio-assay of stem extract and fractions of *Cissampelos pareira* against *Aphis craccivora*.

Extracts and Fractions	LC_50_ (mg/L)	95% Confidence Limits (mg/L)	Slope ± SE	Chi Square	*p* Value
Parent extract (72 h)	5929.00	4268.10–9746.45	1.69 ± 0.30	0.98	0.80
Parent extract (96 h)	4044.83	3137.45–5488.08	2.13 ± 0.32	2.98	0.39
*n*-Hexane fraction (72 h)	1466.98	1045.44–1922.93	1.97 ± 0.31	4.08	0.25
*n*-Hexane fraction (96 h)	1246.92	973.36–1538.01	2.91 ± 0.44	1.05	0.79
Ethyl acetate fraction (72 h)	5534.27	4106.25–8446.65	1.85 ± 0.32	0.31	0.96
Ethyl acetate fraction (96 h)	3992.4	3056.7–5525.54	1.99 ± 0.31	3.48	0.32
*n*-Butanol fraction (72 h)	7766.81	5508.44–13759.15	1.79 ± 0.34	0.36	0.95
*n*-Butanol fraction (96 h)	3840.96	2929.06–5311.91	1.94 ± 0.31	1.53	0.67
Water fraction (72 h)	5861.38	4221.04–9624.86	1.66 ± 0.31	2.30	0.51
Water fraction (96 h)	3761.37	2789.14–5399.91	1.72 ± 0.29	2.16	0.54
Neem Baan (0.15 EC) (72 h)	2587.32	1914.19–3944.86	1.52 ± 0.25	0.71	0.87
Neem Baan (0.15 EC) (96 h)	1206.44	953.26–1528.79	1.98 ± 0.26	3.90	0.26

*n* = 150 insects; no. of replications-3; LC_50_ = lethal concentration to kill 50% of test insect; LC_50_ was calculated for extract and fractions showing >50% mortality using probit analysis.

**Table 5:** Bio-assay of *Cissampelos pareira* pure molecules against *Aphis craccivora*.

Pure Molecules	LC_50_ (mg/L)	95% Confidence Limits (mg/L)	Slope ± SE	Chi Square	*p* Value
Cissamine (48 h)	2744.95	1938.88–4672.36	1.53 ± 0.29	2.34	0.50
Cissamine (72 h)	1556.31	1224.72–2014.71	2.29 ± 0.33	3.17	0.37
Pareirarine (48 h)	1860.57	1423.55–2550.05	1.97 ± 0.31	0.61	0.89
Pareirarine (72 h)	1491.93	1154.87–1962.72	2.10 ± 0.31	1.33	0.72
Curine (48 h)	–	–	–	–	–
Curine (72 h)	3802.47	2656.20–6989.20	1.67 ± 0.32	0.91	0.82
Neem Baan (0.15 EC) (72 h)	2587.32	1914.19–3944.86	1.52 ± 0.25	0.71	0.87
Neem Baan (0.15 EC) (96 h)	1206.44	953.26 –1528.79	1.98 ± 0.26	3.90	0.26

*n* = 150 insects; no. of replications-3; LC_50_ = lethal concentration to kill 50% of test insect; LC_50_ was calculated for extract and fractions using probit analysis; ‘–’ LC_50_ values are not calculated in treatments which showed <50% mortality in the higher concentration.

## Data Availability

Not applicable.

## Supplementary Materials

The data presented in this study are available in the supplementary material, Figure S1a: ^1^H-NMR of curine (1), Figure S1b: ^13^C-NMR of curine (1), Figure S2a: ^1^H-NMR of pareirarine formate (2), Figure S2b: ^13^C-NMR of pareirarine formate (2), Figure S3a: ^1^H-NMR of cissamine (3), Figure S3b: ^13^C-NMR of cissamine (3), Figure S4: Per cent mortality of parent extract and fractions of *C. pareira* root against *A. craccivora,* Figure S5: Per cent mortality of parent extract and fractions of *C. pareira* stem against *A. craccivora,* Figure S6: Per cent mortality of pure molecules of *C. pareira* against *A. craccivora,* Figure S7a: UPLC-DAD chromatograms for standard compounds, Figure S7b: UPLC-DAD chromatograms for extract and fractions of root, Figure S7c: UPLC-DAD chromatograms for extract and fractions of stem, Figure S7d: UPLC-DAD chromatograms for decoction, Figure S8: GC-MS chromatograms for *n*-hexane fractions of root and stem, Table S1: ^1^H and ^13^C-NMR (600 and 150 MHz) data of curine (1) in CD_3_OD + CD_3_COOD, Table S2: ^1^H and ^13^C-NMR (600 and 150 MHz) data of pareirarine formate (2) in CD_3_OD, Table S3: ^1^H and ^13^C-NMR (600 and 150 MHz) data of cissamine (3) in CD_3_OD.
