# Supplementary material for: Insecticidal Activity of Extracts, Fractions, and Pure Molecules of Cissampelos pareira Linnaeus against Aphid, Aphis craccivora Koch
Source: Molecules. 2022 Jan 19;27(3):633. doi: 10.3390/molecules27030633 (PMC8838119; doi:10.3390/molecules27030633)
Supplement: Supplementary file 1 [file molecules-27-00633-s001.zip › molecules-1513331 supplementary.pdf]

Supplementary Materials

Insecticidal Activity of Extracts, Fractions, and Pure Molecules of *Cissampelos pareira* Linnaeus against Aphid, *Aphis craccivora* Koch

Surekha Kumari [1,2,†], Shudh Kirti Dolma [2,3,†], Anmol [1,2], Upendra Sharma [1,2,*] and S. G. Eswara Reddy [2,3,*]

[1] Chemical Technology Division, CSIR-Institute of Himalayan Bioresource Technology, Palampur 176061, Himachal Pradesh, India; surekhakumari46@gmail.com (S.K.); sanmol472@gmail.com (A.)
[2] Academy of Scientific and Innovative Research (AcSIR), CSIR-HRDC Campus, Ghaziabad 201002, Uttar Pradesh, India; skdolma@gmail.com
[3] Entomology Laboratory, Agrotechnology Division, CSIR-Institute of Himalayan Bioresource Technology, Palampur 176061, Himachal Pradesh, India
* Correspondence: ereddy2001@yahoo.com or ereddy@ihbt.res.in (S.G.E.R.); upendraihbt@gmail.com or upendra@ihbt.res.in (U.S.)
† Equal Contribution.

Contents

Figure S1a. ^{1}H NMR of curine (**1**)

Figure S1b. ^{13}C NMR of curine (**1**)

Figure S2a. ^{1}H NMR of pareirarine formate (**2**)

Figure S2b. ^{13}C NMR of pareirarine formate (**2**)

Figure S3a. ^{1}H NMR of cissamine (**3**)

Figure S3b. ^{13}C NMR of cissamine (**3**)

Figure S4. Per cent mortality of parent extract and fractions of *Cissampelos pareira* root against *Aphis craccivora*; The same letters in the error bars (Mean ± SE) within the figure are not statistically different by Tukey's HSD ($p \leq 0.05$)

Figure S5. Per cent mortality of parent extract and fractions of *Cissampelos pareira* stem against *Aphis craccivora*; The same letters in the error bars (Mean ± SE) within the figure are not statistically different by Tukey's HSD ($p \leq 0.05$)

Figure S6. Per cent mortality of pure molecules of *Cissampelos pareira* against *Aphis craccivora*; The same letters in the error bars (Mean ± SE) within the figure are not statistically different by Tukey's HSD ($p \leq 0.05$)

Figure S7a. UPLC-DAD chromatograms for standard compounds

Figure S7b. UPLC-DAD chromatograms for extract and fractions of root

Figure S7c. UPLC-DAD chromatograms for extract and fractions of stem

Figure S7d. UPLC-DAD chromatograms for decoction

Figure S8. GC-MS chromatograms for *n*-hexane fractions of root and stem

Table S1. ^{1}H and ^{13}C NMR (600 and 150 MHz) data of curine (**1**) in CD_3OD + CD_3COOD

Table S2. ^{1}H and ^{13}C NMR (600 and 150 MHz) data of pareirarine formate (**2**) in CD_3OD

Table S3. ^{1}H and ^{13}C NMR (600 and 150 MHz) data of cissamine (**3**) in CD_3OD

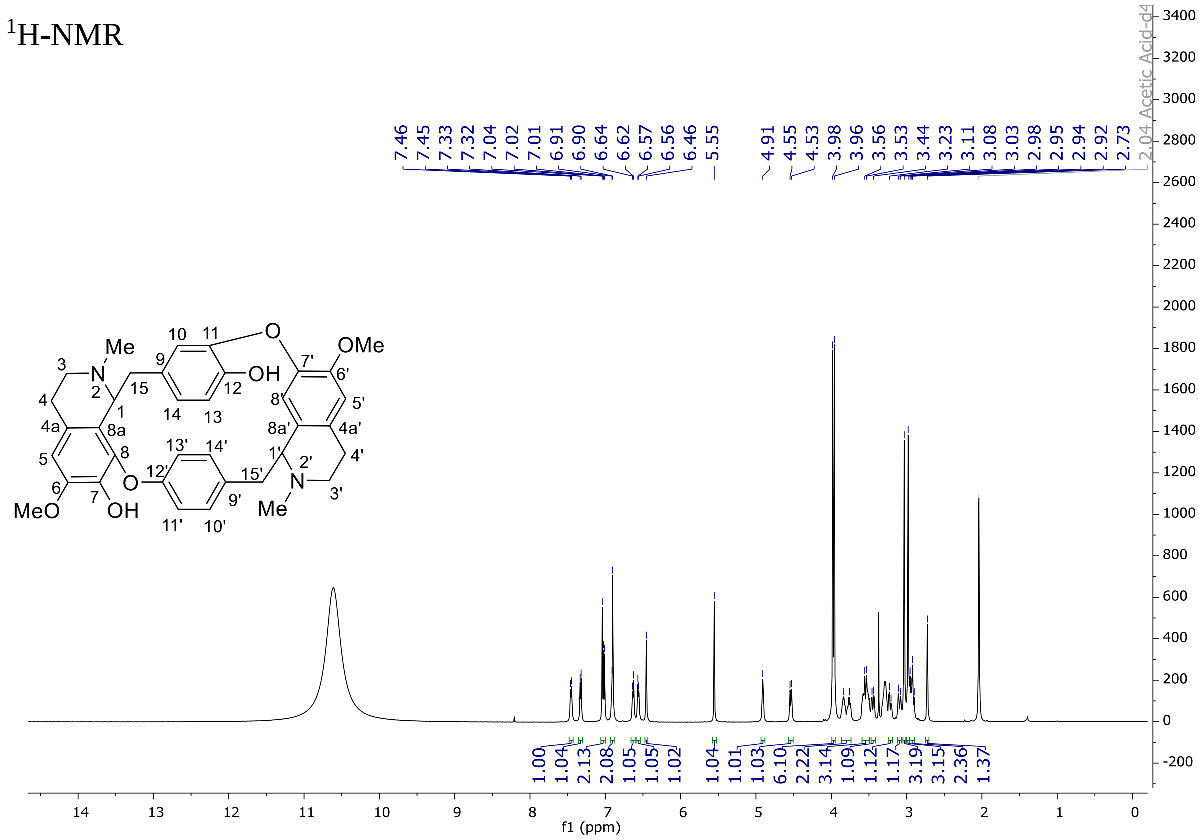

Figure S1a. ^{1}H-NMR spectrum of curine

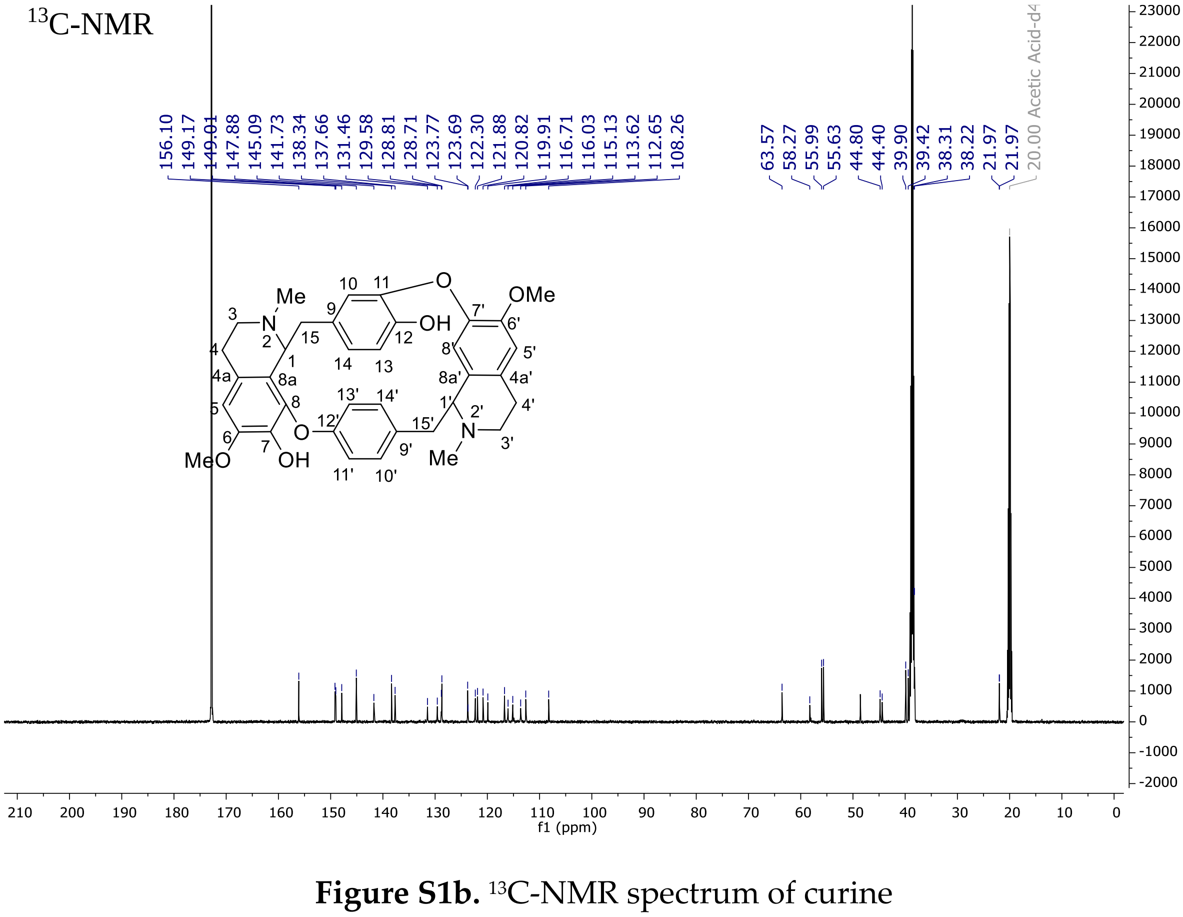

Figure S1b. ^{13}C-NMR spectrum of curine

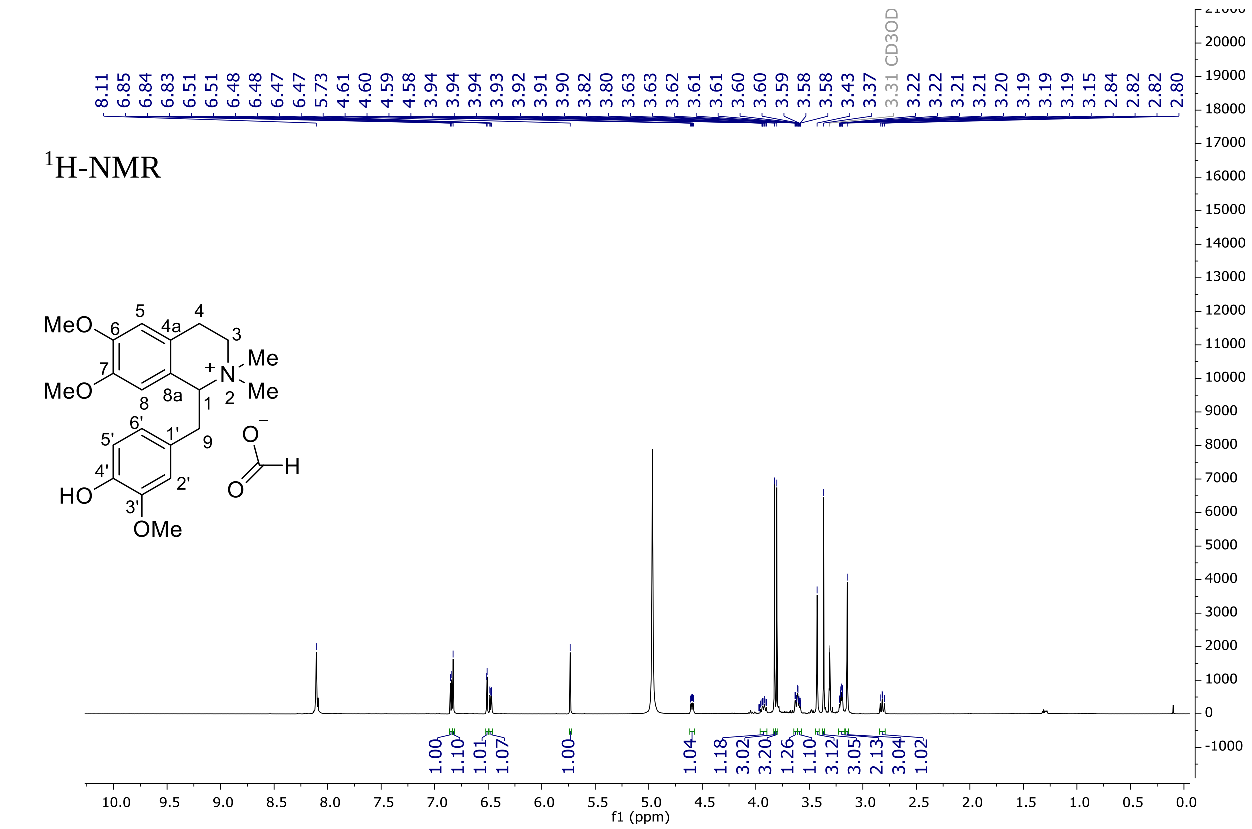

Figure S2a. ^{1}H-NMR spectrum of pareirarine formate

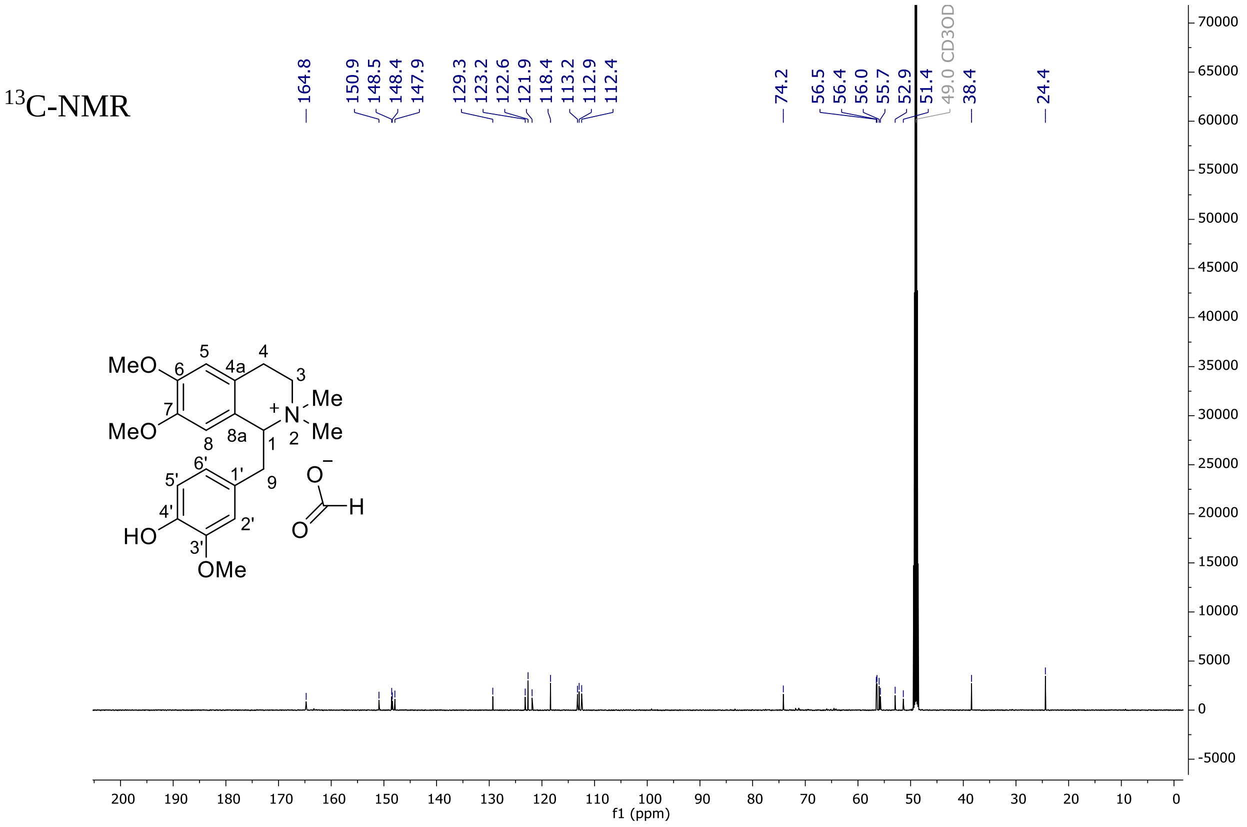

Figure S2b. ^{13}C-NMR spectrum of pareirarine formate

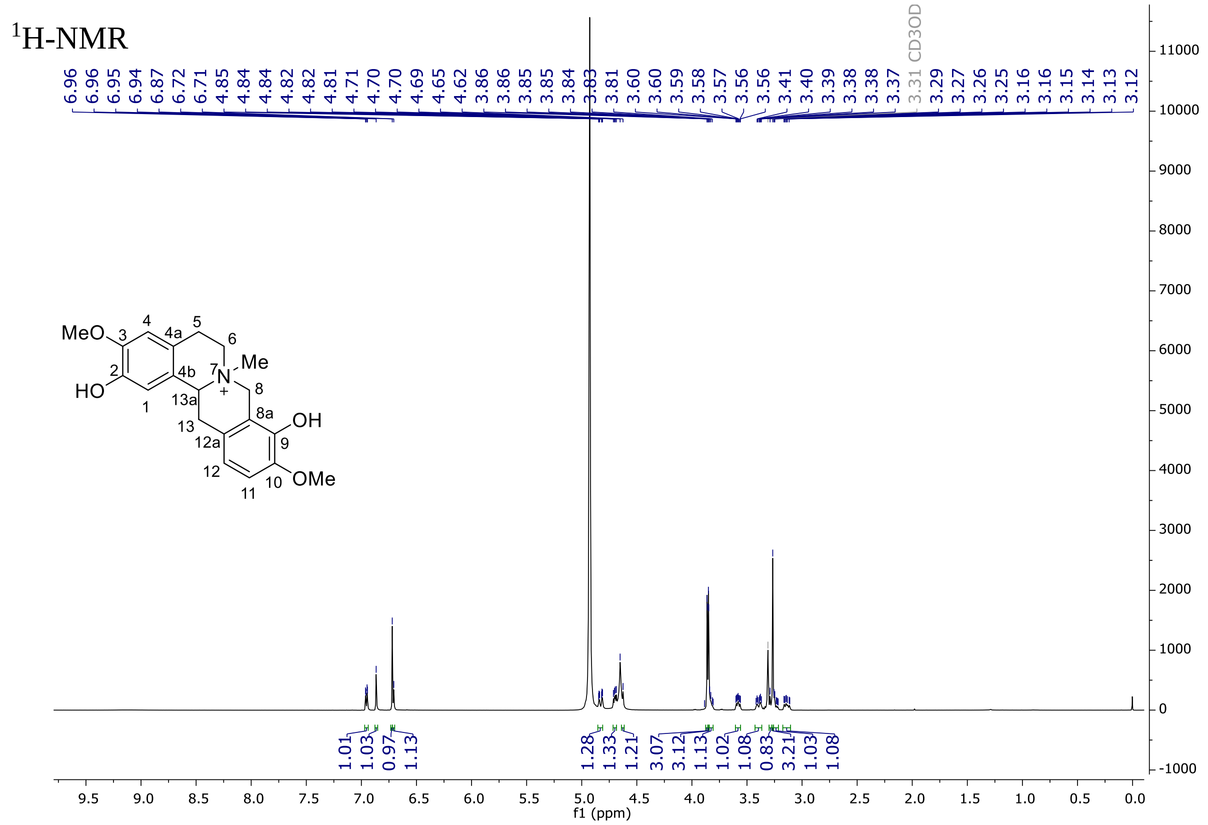

Figure S3 a. ^{1}H-NMR spectrum of cissamine

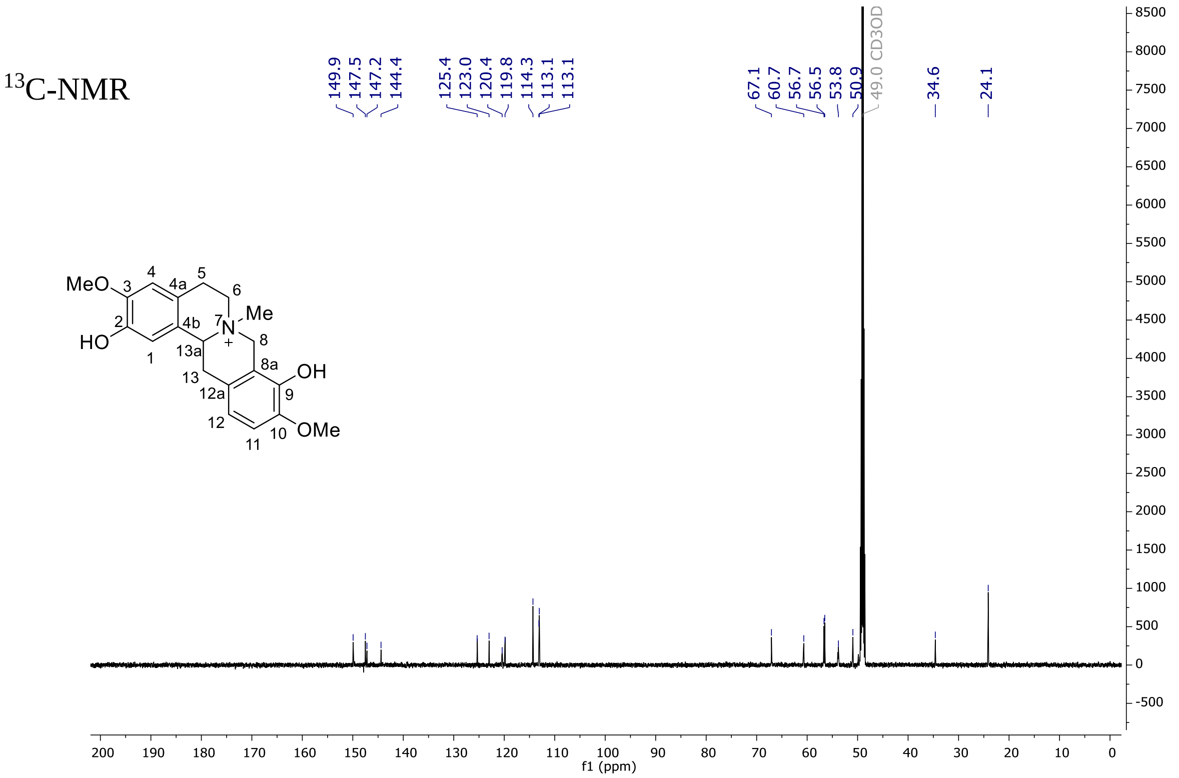

Figure S3b. ^{13}C-NMR spectrum of cissamine

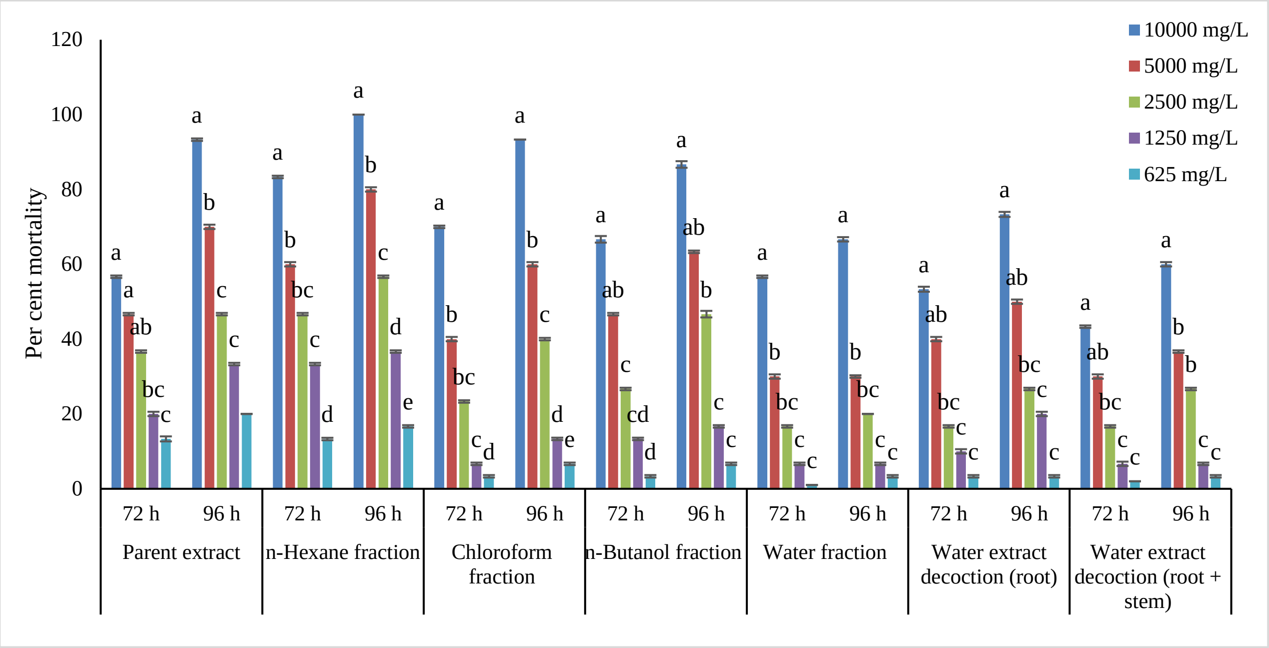

Figure S4. Per cent mortality of parent extract and fractions of *Cissampelos pareira* root against *Aphis craccivora*; The same letters in the error bars (Mean ± SE) within the figure are not statistically different by Tukey's HSD (p ≤ 0.05)

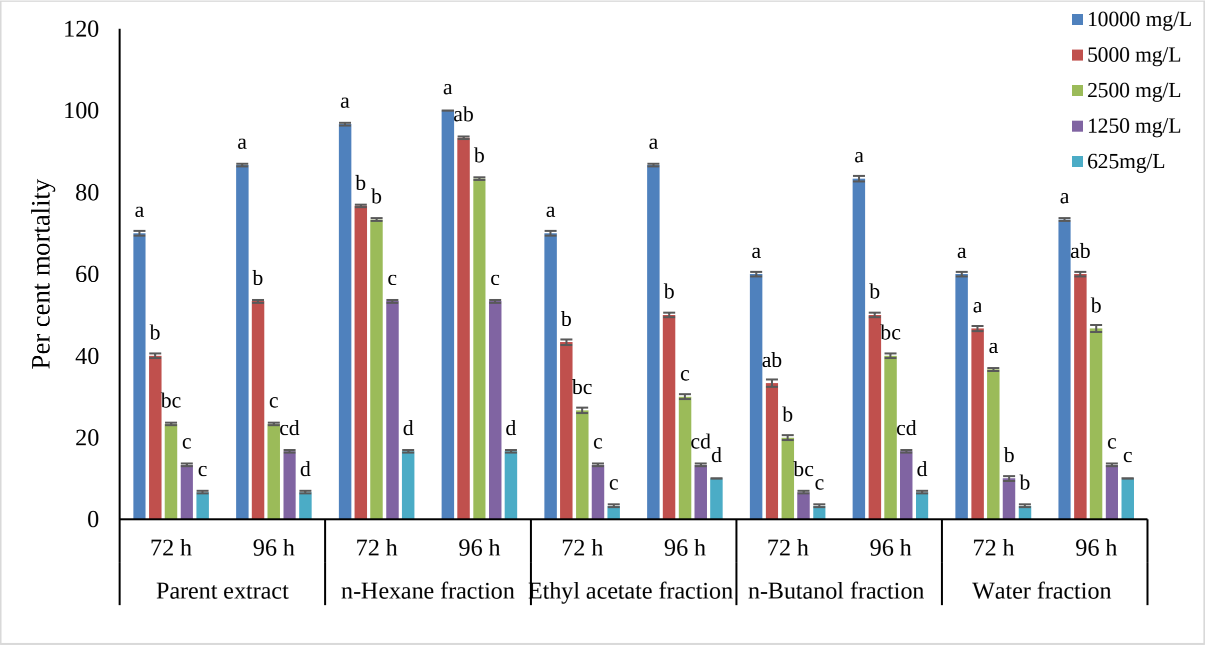

Figure S5. Per cent mortality of parent extract and fractions of *Cissampelos pareira* stem against *Aphis craccivora*; The same letters in the error bars (Mean ± SE) within the figure are not statistically different by Tukey's HSD ($p \leq 0.05$)

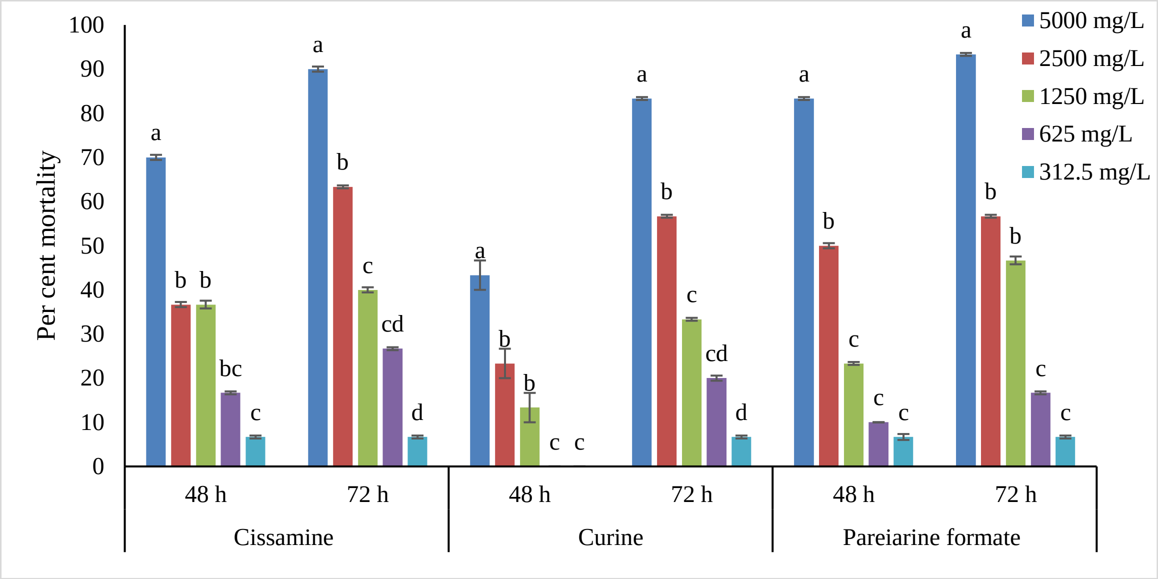

Figure S6. Per cent mortality of pure molecules of *Cissampelos pareira* against *Aphis craccivora*; The same letters in the error bars (Mean ± SE) within the figure are not statistically different by Tukey's HSD ($p \leq 0.05$)

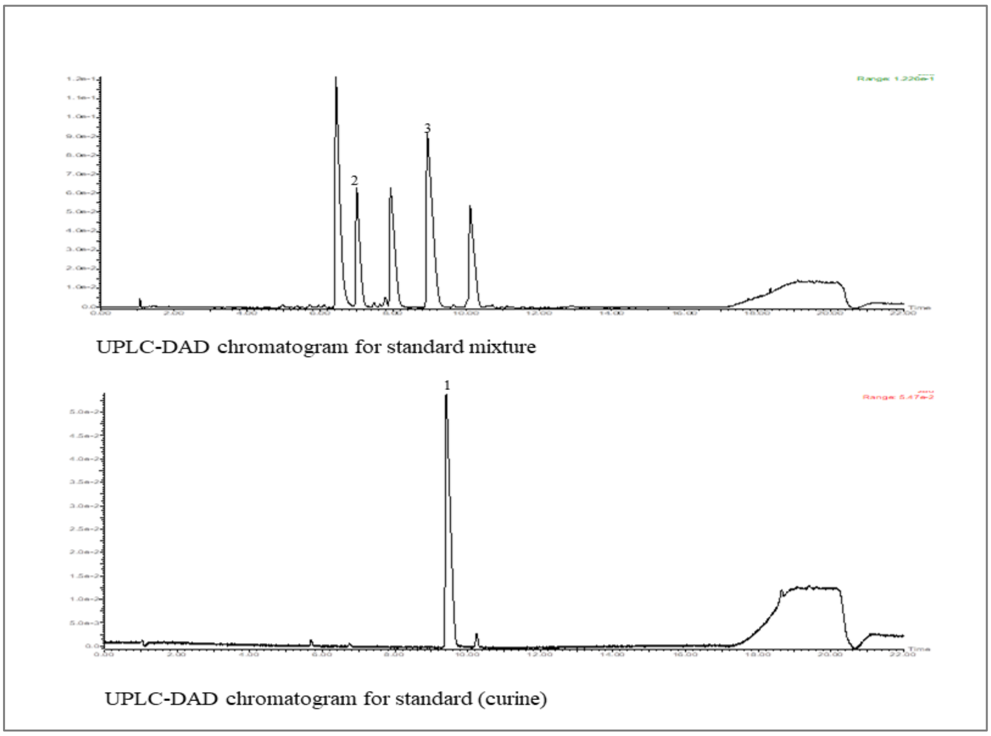

Figure S7a. UPLC-DAD chromatograms for standard compounds

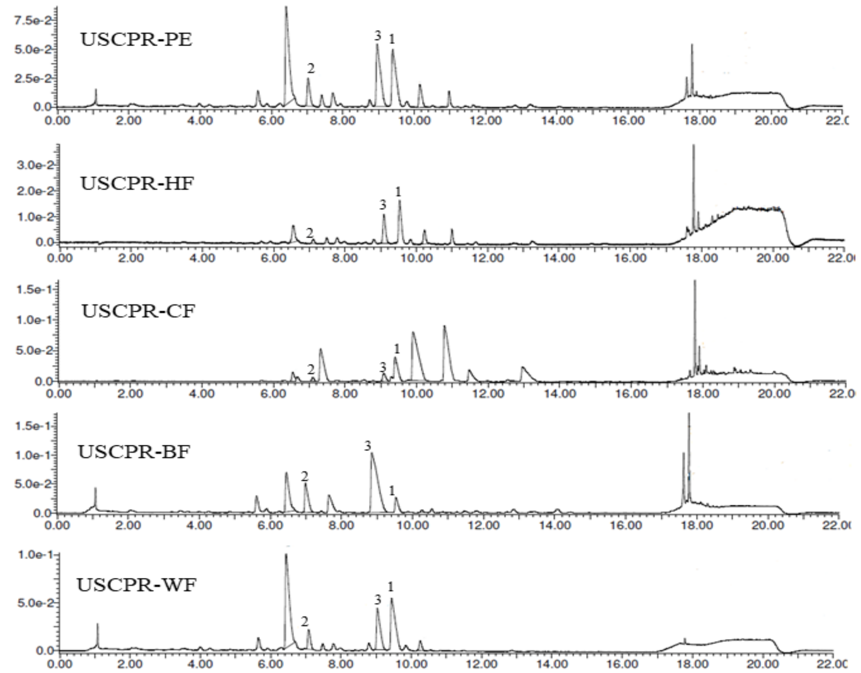

USCPR-PE: parent root extract; USCPR-HF: *n*-hexane fraction of root; USCPR-CF; chloroform fraction of root; USCPR-BF: *n*-butanol fraction of root; USCPR-WF: water fraction of root

Figure S7b. UPLC-DAD chromatograms for extract and fractions of root

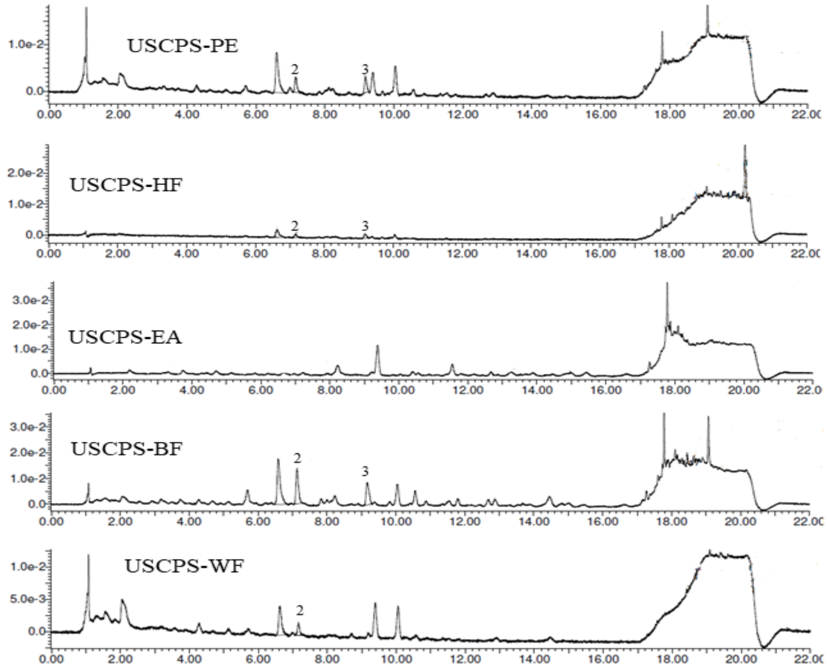

USCPS-PE: parent stem extract; USCPS-HF: *n*-hexane fraction of stem; USCPS-EA: ethyl acetate fraction of stem; USCPS-BF: *n*-butanol fraction of stem: USCPS-WF: water fraction of stem

Figure S7c. UPLC-DAD chromatograms for extract and fractions of stem

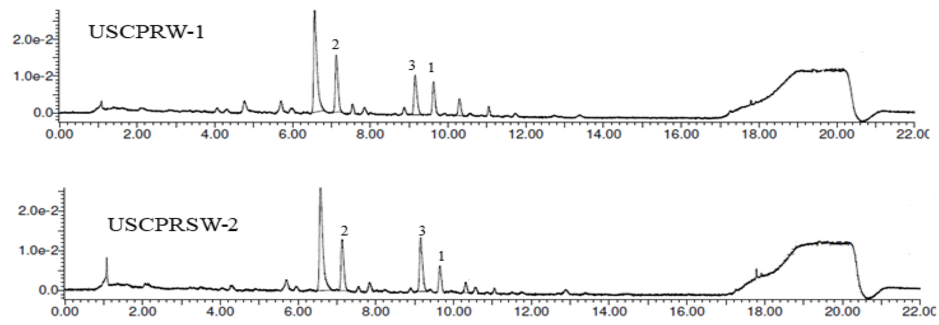

USCPRW-1: root decoction; USCPRSW-2: root and stem decoction

Figure S7d. UPLC-DAD chromatograms for decoction

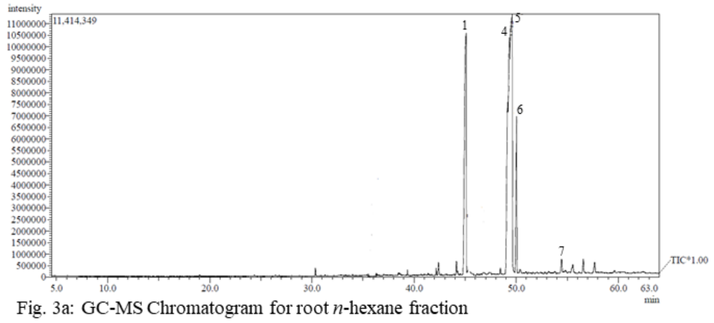

Fig. 3a: GC-MS Chromatogram for root *n*-hexane fraction

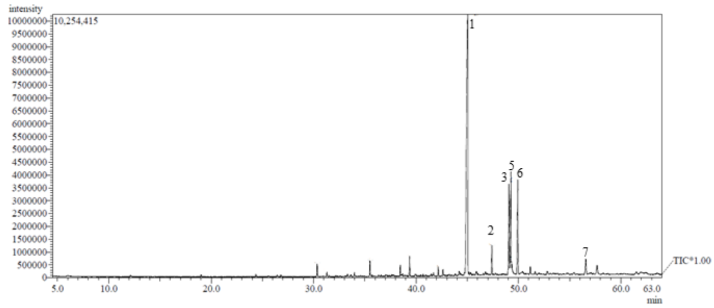

Fig. 3b: GC-MS Chromatogram for stem *n*-hexane fraction

Figure S8. GC-MS chromatograms for *n*-hexane fractions of root and stem.

Table S1. ^{1}H and ^{13}C NMR (600 and 150 MHz) data of curine (**1**) in CD_3OD + CD_3COOD

Positions	δ_C	δ_H	Positions	δ_C	δ_H
1	58.2	4.91 (s)	1'	63.5	4.54 (d)
2 (N-CH_3)	39.9	2.98 (s)	2' (N-CH_3)	39.4	3.03 (s)
3	44.8	3.46-3.44 (m); 2.95-2.90 (m)	3'	44.4	3.83 (m); 3.56-3.53 (m)
4	21.9	2.95-2.90 (m);	4'	21.9	3.26-3.21 (m); 3.11-3.08 (m)
4a	119.9	-	4a'	123.6	-
5	108.2	7.05 (s)	5'	112.6	6.90 (s)
6	149.0	-	6'	149.1	-
7	137.6	-	7'	145.0	-
8	138.3	-	8'	115.2	5.55 (s)
8a	120.8	-	8a'	121.8	-
9	128.7	-	9'	128.8	-
10	122.3	6.46 (s)	10'	131.4	6.63 (d, *J* = 7.9 Hz)
11	141.7	-	11'	113.6	6.56 (d, *J* = 6.9 Hz)
12	147.8	-	12'	156.1	-
13	116.7	7.02 (d, *J* = 8.3 Hz)	13'	116.0	6.91 (m)[a]
14	123.7	7.32 (d, *J* = 7.7 Hz)	14'	129.5	7.45 (d, *J* = 8.0 Hz)
15	38.3	3.46-3.44 (m); 2.95-2.90 (m)	15'	38.2	3.56-3.54 (m)
6-OCH_3	55.9	3.98 (s)	6'-OCH_3	55.6	3.96 (s)

a = overlapped signal

Table S2. ^{1}H and ^{13}C NMR (600 and 150 MHz) data of pareirarine formate (**2**) in CD_3OD

Positions	δ_C	δ_H
1	74.1	4.60 (dd)
2 (N-CH_3)	52.9	3.43 (s)
2 (N-CH_3)	51.3	3.15 (s)
3	55.7	3.90 -3.97 (m);<br>3.61- 3.63 (m)
4	24.4	3.19-3.22 (m)
4a	121.8	-
5	112.4	6.83 (s)
6	150.9	-
7	147.1	-
8	113.2	5.73 (s)
8a	123.1	-
9	38.4	3.58 -3.60 (m);<br>2.80-2.84 (m)
1'	129.3	-
2'	118.3	6.51 (d, J = 2.1 Hz)
3'	148.4	-
4'	148.5	-
5'	112.9	6.85 (d, J = 8.2 Hz)
6'	122.6	6.48 (dd, J = 8.2, 2.1 Hz)
6, 7, 3' (OCH_3)	56.5, 56.3, 55.9	3.82 (s), 3.80 (s), 3.37 (s)

Table S3. ^{1}H and ^{13}C NMR (600 and 150 MHz) data of cissamine (**3**) in CD_3OD

Positions	δ_C	δ_H
1	114.3	6.66 (s)
2	147.2	-
3	149.9	-
4	113.1	6.81 (s)
4a	120.4	-
4b	125.3	-
5	24.1	3.17-3.20 (m)
6	53.8	3.50-3.55 (m), 3.75-3.78 (m)
7 (N-CH_3)	50.9	3.21 (s)
8	60.6	4.75-4.79 (m), 4.57 (m)
8a	120.4	-
9	144.4	-
10	147.5	-
11	113.0	6.90 (d, *J* = 8.4, 2.0 Hz)
12	119.8	6.65 (d)
12a	123.3	-
13	34.6	3.30-3.37 (m), 3.05-3.10 (m)
13a	67.0	4.64 (dd)
3, 10 (OCH_3)	56.6, 56.5	3.80 (s), 3.79 (s)
